# Collective cell mechanics of epithelial shells with organoid-like morphologies

**DOI:** 10.1038/s41467-020-17535-4

**Published:** 2020-07-30

**Authors:** Jan Rozman, Matej Krajnc, Primož Ziherl

**Affiliations:** 1 Jožef Stefan Institute, Jamova 39, Ljubljana, 1000 Slovenia; 2 Faculty of Mathematics and Physics, University of Ljubljana, Jadranska 19, Ljubljana, 1000 Slovenia; 3 Lewis–Sigler Institute for Integrative Genomics, Princeton University, Washington Road, Princeton, NJ 08540 USA

**Keywords:** Computational biophysics, Biological physics

## Abstract

The study of organoids, artificially grown cell aggregates with the functionality and small-scale anatomy of real organs, is one of the most active areas of research in biology and biophysics, yet the basic physical origins of their different morphologies remain poorly understood. Here, we propose a mechanistic theory of epithelial shells which resemble small-organoid morphologies. Using a 3D surface tension-based vertex model, we reproduce the characteristic shapes from branched and budded to invaginated structures. We find that the formation of branched morphologies relies strongly on junctional activity, enabling temporary aggregations of topological defects in cell packing. To elucidate our numerical results, we develop an effective elasticity theory, which allows one to estimate the apico-basal polarity from the tissue-scale modulation of cell height. Our work provides a generic interpretation of the observed epithelial shell morphologies, highlighting the role of physical factors such as differential surface tension, cell rearrangements, and tissue growth.

## Abstract (web summary)

The physical origins of man-made tissue morphologies with organ-like microscopic anatomy and functionality remain poorly understood. Here, authors propose a mechanistic theory of these structures, employing a 3D vertex model to reproduce the characteristic morphologies such as branched shapes.

## Introduction

In the past decade, organoids became one of the most interesting topics in cells and tissue biology, organogenesis, developmental biology, and study of disease^1,2^. Organoids are aggregates of cells grown in vitro so as to form miniature replicas of a given organ such as kidney, lung, and brain^3,4^. In most cases, they consist of a closed shell of sheet-like tissue enclosing a lumen, and they have the same microscopic morphology as the mimicked organ itself, e.g., the villus-crypt pattern of the mammalian intestine. Organoids are often grown from embryonic stem cells driven so as to develop a given tissue identity and cultured in a medium such as Matrigel. They can also be grown from adult tissue- or stem cells^5,6^.

One of the defining features of organoids is their form. Their initially simple shape transforms into a given morphology as cells divide and mature^7^. The most common organoid morphologies include budded and branched shapes with spherical or finger-like protrusions, respectively; some branched shapes develop bifurcating networks. Organoid shape depends on many factors and processes including the intrinsic physical features of individual cells, cell growth and division rates, cell differentiation, etc. So far, theoretical studies of the role of these factors were mostly carried out by representing the tissue as a continuous concentration field in a model of Cahn–Hilliard type^8,9^ or as an ensemble of spherical entities using discrete models^7,10^. These approaches account for the biochemical regulation of organoid growth, yielding invaluable insight into, e.g., strategies of anticancer therapy^9^. Recently, a 3D vertex model was employed to study how chemical patterning controlling the local cell-growth rate may feed back to the mechanics to determine organoid morphology^11^. Together with results addressing transformations of epithelial shells^12^, these insights demonstrate that many features of the observed organoid shapes can be interpreted using physical models based on mechanisms arising from the underlying cell- and tissue-level biological processes.

Here we enhance this perspective by analyzing a surface tension-based vertex model of single-cell-thick epithelial shells. To emphasize the collective mechanical effects, we study tissues consisting of cells of identical properties; in this respect, they differ from organoids and resemble tumor and other types of spheroids where cell differentiation is often absent. Our shells are also devoid of any biological function of the organoids, but their spatial structure with a single layer of cells enclosing the lumen is organoid-like. The diversity of the obtained shapes is striking, especially since their formation relies exclusively on non-specific cell-scale mechanics such as the apico-basal differential surface tension.

Our simple model reproduces many observed organoid morphologies and is interpreted in terms of a theory of elasticity, which contains several interesting elements such as curvature-thickness coupling. Furthermore, we explore the formation of in-plane cell arrangements and find that topological defects in cell packing induced by active rearrangements can act as seeds for branching morphogenesis, leading to out-of-equilibrium branched shapes. We also study the shape formation during tissue growth through successive cell divisions. Overall, these results reveal the generic mechanisms of morphogenesis of small epithelial shells of identical cells.

## Results

### Active vertex model

**Fig. 1:**
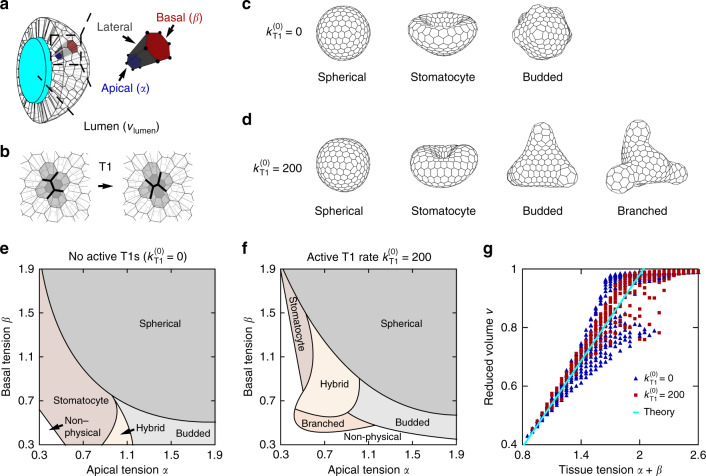
**Mechanical model predicts several characteristic morphologies**. **a** Schematics of the model epithelial shell with a lumen of volume *v*_lumen_ (expressed in units of cell volume *V*_cell_) indicated in cyan, and a cell with apical, lateral, and basal sides; also indicated are the dimensionless apical and basal tensions *α* and *β*, respectively. **b** T1 transition. **c** Representative shapes with *N*_c_ = 300 cells, *v*_lumen_ = 100, and ${k}_{{\rm{T}}1}^{(0)}=0$: spherical (*α* = 1.2, *β* = 1.2), stomatocyte (*α* = 0.7, *β* = 0.5), and budded (*α* = 1.5, *β* = 0.3) morphologies. Panel **d** shows model epithelial shells at the same *N*_c_ and *v*_lumen_ but with ${k}_{{\rm{T}}1}^{(0)}=200$: spherical (*α* = 1.2, *β* = 1.2), stomatocyte (*α* = 0.5, *β* = 1.1), budded (*α* = 1.1, *β* = 0.5), and branched (*α* = 0.7, *β* = 0.5) morphologies. **e** Phase diagram of the *N*_c_ = 300, *v*_lumen_ = 100, and ${k}_{{\rm{T}}1}^{(0)}=0$ shapes in the (*α*, *β*) plane. **f** Phase diagram at the same *N*_c_ and *v*_lumen_ as in panel e but with ${k}_{{\rm{T}}1}^{(0)}=200$. **g** Reduced volume *v* vs. tissue tension *α* + *β*. The solid cyan line is a theoretical prediction of the relation in non-spherical shapes [Eq. (3)].

We theoretically study the shapes of single-cell-thick epithelial shells encompassing an incompressible fluid-like interior referred to as lumen (Fig. 1a). The cells too are assumed to be incompressible, and they carry a surface energy with three distinct tensions at the inner apical side, the outer basal side, and the lateral sides where they adhere to their neighbors^13^. By considering surface energy alone, we disregard several elements of cell mechanics such as the acto-myosin cable at the apical surface, which allows us to work with fewer model parameters. We further assume that all cells are identical in terms of their surface tensions and cell volumes *V*_cell_, and we explore a generic scenario where complex morphologies arise directly from an interplay between the preferred shape of individual cells and long-range interactions due to lumen incompressibility rather than from a specific spatial variation of cell properties.

We use a 3D vertex model^14^ where cells are represented by polyhedra with polygonal apical and basal sides and rectangular lateral sides (Fig. 1a); the topologies of the apical and the basal cell networks are identical. Cell shapes are parametrized by vertex positions **r**_*j*_ = (*x*_*j*_, *y*_*j*_, *z*_*j*_), where *x*_*j*_, *y*_*j*_,  and *z*_*j*_ are dimensionless coordinates expressed in units of ${V}_{{\rm{cell}}}^{1/3}$. The dimensionless energy of the shell expressed in units of ${\Gamma }_{{\rm{l}}}{V}_{{\rm{cell}}}^{2/3}$ (where Γ_l_ is the surface tension of the lateral sides) is given by a sum over all *N*_c_ cells:

1 $$w=\mathop{\sum }\limits_{i = 1}^{{N}_{{\rm{c}}}}\left[\alpha {a}_{{\rm{a}}}^{(i)}+\beta {a}_{{\rm{b}}}^{(i)}+\frac{1}{2}{a}_{{\rm{l}}}^{(i)}\right].$$

Here *α* and *β* are the dimensionless tensions of the apical and basal sides, respectively, expressed in units of Γ_l_ (Fig. 1a), whereas ${a}_{{\rm{a}}}^{(i)},{a}_{{\rm{b}}}^{(i)},$ and ${a}_{{\rm{l}}}^{(i)}$ are the areas of the apical, basal, and lateral sides of the *i*th cell, respectively, expressed in units of ${V}_{{\rm{cell}}}^{2/3}$.

In our model, tissue dynamics includes two processes: (i) deterministic relaxation of the system along the energy gradient and (ii) cell rearrangements driven by active junctional noise, which fluidize the model tissue. During each time step, the vertices are first moved according to the overdamped equation of motion d**r**_*j*_/d*t* = −∇_*j*_*w*; here ∇_*j*_ is the gradient with respect to the dimensionless **r**_*j*_, *w* is the dimensionless energy, and dimensionless time *t* is measured in units of ${\tau }_{0}={({\Gamma }_{{\rm{l}}}\mu )}^{-1}$ with *μ* being vertex mobility. Additionally, cells are allowed to rearrange through T1 transitions (Fig. 1b; see “Methods” section) like in ref. ^14^. In particular, active noise at cell–cell junctions due to stochastic turnover dynamics of molecular motors provides energy fluctuations which can drive the system from one metastable state to another via T1 transitions. These transitions are implemented by swapping the four cells arranged around a given lateral side, which is the physical cell–cell junction.

It is convenient to view the junction projected onto the midplane between the apical and the basal surface—and to treat it as an edge of length halfway between those of the corresponding edges on the apical and the basal surface. Since the thus-redefined cell–cell junctions that have a longer length are associated with a higher energy barrier between the two metastable states, they are less likely to undergo the T1 transition. To describe this, we employ the threshold-based scheme where the probability of a T1 transition in junctions shorter than the threshold *δl* is unity, whereas junctions longer than the threshold undergo the transition with a probability ${k}_{{\rm{T}}1}\delta t/{\mathcal{E}}$^14^. Here *k*_T1_ is the rate of active T1 transitions measured in units of ${\tau }_{0}^{-1}$, *δt* = 10^−4^ is the time step, and ${\mathcal{E}}$ is the number of cell–cell junctions. We choose *δl* = 0.15 (defined as a dimensionless quantity expressed in units of ${V}_{{\rm{cell}}}^{1/3}$), which is between a quarter and a third of the average junction length; the results do not depend significantly on this choice (Supplementary Fig. 4b). As shown previously, tissues with this type of noise behave like viscous fluids with activity-dependent stress-relaxation time scale and the associated effective viscosity^14^. Therefore, an important aspect of the shapes of our epithelial shells is their sensitivity to the active T1 rate *k*_T1_.

In most cases discussed here, the model parameters include only a handful of dimensionless quantities: the apical and basal surface tensions *α* and *β*, the volume of the lumen relative to the cell volume *v*_lumen_ = *V*_lumen_/*V*_cell_, the cell number *N*_c_, and at most two parameters related to junctional activity.

### Phase diagram

We start by studying shells with fixed cell number *N*_c_ = 300 and lumen volume *v*_lumen_ = 100 and we first consider the case with no active T1 transitions; this limit is denoted by ${k}_{{\rm{T}}1}^{(0)}=0$ for consistency of notation, with the meaning of ${k}_{{\rm{T}}1}^{(0)}$ explained in the next paragraph. The initially spherical shape evolves into three characteristic types of morphologies depending on *α* and *β*: stomatocyte (cup-like), budded, and spherical (Fig. 1c; Supplementary Movie 1). In the phase diagram in the (*α*, *β*) plane (Fig. 1e; Supplementary Fig. 1a), spherical shapes occupy the region where the tissue tension defined by *α* + *β* is larger than about 1.9, budded shapes emerge at *α* larger than about 1 and *β* smaller than about 0.6, whereas stomatocytes occur in the region where *α* + *β* is smaller than 1.9 but larger than around 0.9 and *α* is less than about 1. In many shapes, the characteristic features are well-developed so that the classification is unambiguous, but this is not always the case. For example, a generally round shell with small buds may be regarded either as a spherical or as a budded shape. Shapes found at the boundary between the stomatocyte and the budded-shell domain contain features of both morphologies and are best referred to as hybrid (Supplementary Fig. 1c). Finally, at tissue tensions *α* + *β* that are smaller than about 0.9 we find non-physical self-overlapping shapes.

We next analyze the case where relaxation is accompanied by an initially high rate of active cell rearrangements with ${k}_{{\rm{T}}1}(t=0)={k}_{{\rm{T}}1}^{(0)}=200$, which decreases to zero as ${k}_{{\rm{T}}1}(t)={k}_{{\rm{T}}1}^{(0)}({t}_{\max }-t)/{t}_{\max }$; here ${t}_{\max }=1000$ is the total simulation time. The finite duration of junctional activity mimics the natural course of morphogenetic events, which, guided by biochemical signals, begin and end at a predefined time; the linear temporal profile is just an example of a gradual decrease of activity. In “Active cell rearrangements” section, we show that this choice does not significantly affect the results. Like in the case with no activity, we obtain the budded, stomatocyte, and spherical shapes. However, the active T1 transitions also give rise to the branched morphologies appearing at the apical and basal tensions somewhat smaller than unity, that is in a part of the domain occupied by stomatocytes if the junctional activity is absent (Fig. 1d, f; Supplementary Figs. 1b and 2, and Supplementary Movie 2). Like before, the phase diagram also contains hybrid shapes that feature either multiple invaginations, both invaginations and evaginations, or are devoid of any clear features (Supplementary Fig. 1d); these shapes appear at *α* ≈ *β* and tissue tension *α* + *β* between about 1 and 2. The domain of non-physical shapes is somewhat larger than in absence of active T1 transitions.

The results obtained using the *N*_c_ = 300 shells are quite representative of our model. This is demonstrated by the four sets of shapes in Supplementary Fig. 3, which show that with an appropriate rescaling of the preferred lumen volume *v*_lumen_ (see “Methods” section), the type of shell morphology does not change significantly with cell number *N*_c_ even if *N*_c_ is halved or doubled. On the other hand, the fixed lumen volume constraint does play an important role in the model morphologies. This constraint is implemented using an auxiliary harmonic volumetric energy term, which ensures that the actual lumen volume does not depart from the preferred value. If the modulus *K*_lumen_ of the auxiliary term is small enough compared to *α* and *β* (e.g., 0.001) then the actual and the preferred volumes differ considerably and the shapes lack clear features and are more or less round (Supplementary Fig. 4a).

### Origin of shape

Aside from the role of active T1 transitions discussed in “Active cell rearrangements” section, the main mechanisms responsible for the formation of the distinct morphologies are (i) the incompatibility of the preferred surface area of the shell and the enclosed lumen volume and (ii) the apico-basal differential tension. In particular, the preferred cell width-to-height ratio scales with the tissue tension *α* + *β* as  ~ (*α* + *β*)^−1^ so that small *α* + *β* drive the columnar-to-squamous transition^14^. In turn, this increases the shell surface area at a fixed lumen volume, leading to complex non-spherical shapes. A similar mechanism was previously studied within a 2D model of tissue cross section^15^.

The area-volume incompatibility can be quantified by the reduced volume defined by

2 $$v=\frac{6\sqrt{\pi }V}{{A}^{3/2}}\ .$$

Here *A* is the area of the midplane surface located halfway between the basal and the apical side of the shell and *V* is the corresponding enclosed volume; *v* = 1 for a sphere, and its value decreases with increasing area at constant volume. We compute the reduced volumes of all 1328 shapes used to construct the phase diagrams in Fig. 1e, f, and find that they depend strongly on the tissue tension *α* + *β* (Fig. 1g; Supplementary Fig. 5a). The numerically obtained dependence agrees well with the analytical prediction, where we estimate the shell midplane surface area by considering a flat epithelium of identical cells with regular hexagonal basal and apical sides^14^. From the force balance along cell height *h*, ∂*w*/∂*h* = 0, and the relation between (dimensionless) cell volume 1, height *h*, and area *a*, which reads 1 = *ha*, we can calculate the equilibrium cell height ${h}_{0}=\left({2}^{1/3}/{3}^{1/6}\right){(\alpha +\beta )}^{2/3}$ and midplane area ${a}_{0}=\left({3}^{1/6}/{2}^{1/3}\right){(\alpha +\beta )}^{-2/3}$. Inserting the expression for *a*_0_ into Eq. (2) yields

3 $$v=\frac{{2}^{3/2}{3}^{3/4}\sqrt{\pi }{v}_{{\rm{midplane}}}}{{N}_{{\rm{c}}}^{3/2}}\left(\alpha +\beta \right),$$

where the volume enclosed by the midplane *v*_midplane_ = 223 (expressed in units of *V*_cell_) is the average taken from the simulated shapes. The relation between *v* and *α* + *β* given by Eq. (3) is plotted in Fig. 1g using solid cyan line.

While the apico-basal differential tension *α* − *β* has a rather limited effect on the reduced volume *v* as witnessed by the limited spread of points in Fig. 1g, it is often crucial for the formation of budded and stomatocyte morphologies. This is because the buds consist of cells with apical sides smaller than basal sides, whereas invaginations in the stomatocyte shapes require cells where apical sides are larger than basal sides. The former cell type is energetically preferred at *α* − *β* > 0, whereas the latter is favored at *α* − *β* < 0.

An insightful way of interpreting the shape of our epithelial shells builds on the analogy with lipid vesicles characterized by spontaneous curvature^16^. In particular, both epithelial shells and vesicles are bodies defined by closed surfaces with a well-defined area and enclosed volume; in addition, epithelia with junctional activity are 2D fluids just like lipid membranes. Finally, the apico-basal polarity due to differential tension *α* − *β* leads to a preferred conical shape of cells^13,17^ so that the tissue has a certain spontaneous curvature reminiscent of that in asymmetric lipid membranes, say due to inclusions. Although the ratio of wall thickness and body size in the epithelial shells is much larger than in vesicles, this comparison is quite illuminating because of both similarities and differences, especially within the context of the theory of elasticity developed in the Curvature-thickness coupling section. As far as the characteristic morphologies are concerned, we note that the phase diagrams of epithelial shells and vesicles both feature stomatocytes at negative spontaneous curvatures and differential tensions *α* − *β*, respectively, whereas the prolate and pear-like vesicles at positive curvatures resemble budded shells found at positive differential tensions (Supplementary Fig. 6).

The shapes shown in Fig. 1d as examples of active shells are characteristic of a given set of model parameters but are not unique due to the stochasticity of cell rearrangements. The resulting variability is illustrated by Supplementary Fig. 5c, which shows three branched shells at *α* = 0.7 and *β* = 0.5. All of these shapes have five unevenly articulated branches of somewhat different lengths and diameters. To explore this variability, we simulate 300 instances at the four pairs of *α* and *β* from Fig. 1d, and we compute the corresponding distributions of reduced volumes. We find that shape variability is largest in the budded shell and smallest in the spherical one (Supplementary Fig. 5d–g). The variability of the budded shape is likely due its location in the phase diagram: it lies close to the boundary between the budded and the branched morphologies, and thus in some instances the shape of the buds may be more pronounced than in others. Nevertheless, throughout the phase diagram the standard deviation of the distribution never exceeds  ≈ 0.04, which shows that the reduced volumes of our epithelial shells are well-defined (Supplementary Fig. 5b). Note that since budded and stomatocyte morphologies appear at similar values of *α* + *β*, their reduced volumes *v* are similar too (Supplementary Fig. 5a).

### Active cell rearrangements

**Fig. 2:**
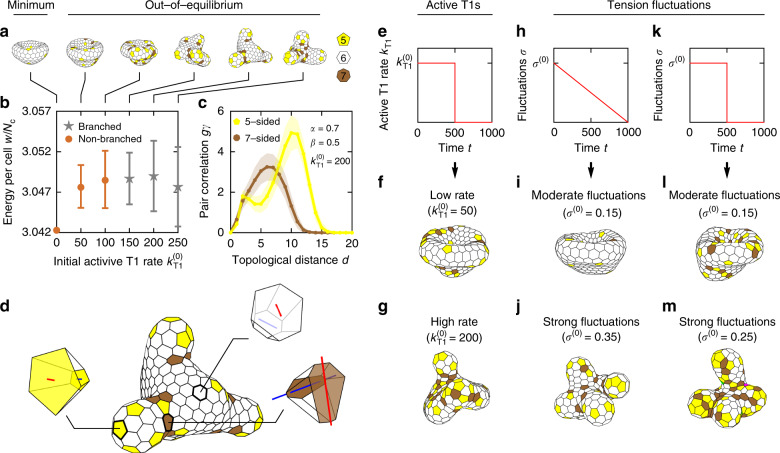
**High rate of active T1 transitions is necessary for formation of branched morphologies**. **a** Sequence of *α* = 0.7, *β* = 0.5, *N*_c_ = 300,  and *v*_lumen_ = 100 shapes with ${k}_{{\rm{T}}1}^{(0)}=0,50,100,150,200,$ and 250. Cells are color-coded according to polygon class; pentagonal cells are yellow, hexagonal are white, and heptagonal are brown. **b** Energy of final epithelial-shell shapes as a function of the initial active T1 rate ${k}_{{\rm{T}}1}^{(0)}$. Symbols and error bars represent the arithmetic mean and the standard deviation of the energies over 100 instances, respectively; at ${k}_{{\rm{T}}1}^{(0)}=0$ the mechanics is completely deterministic and the width of the error bar vanishes. **c** Distributions of topological distances between pairs of pentagons (yellow) and heptagons (brown) in the shell at ${k}_{{\rm{T}}1}^{(0)}=200$ (fifth shape from the left in panel **a**). The symbols show the arithmetic mean of the pair correlation over 300 simulated instances, whereas the shaded areas represent the standard deviation. Solid lines guide the eye. In **d**, the ${k}_{{\rm{T}}1}^{(0)}=200$ shell is magnified and the full 3D shapes of representative 5-, 6-, and 7-coordinated cells are replotted with the long axes of their apical and basal sides indicated by blue and red lines, respectively; line lengths are proportional to the anisometry of the side in question (see “Methods” section). Panels **f** and **g** show the model shapes at ${k}_{{\rm{T}}1}^{(0)}=50$ and 200 in case of a step-like temporal profile of the active T1 rate (**e**). In panels **i** and **j**, we present epithelial shells obtained within a model, where T1 transitions are generated by fluctuations in line tension with a linear temporal profile (**h**), the former for *σ*^(0)^ = 0.15 and the latter for *σ*^(0)^ = 0.35; panels **l** and **m** show shapes obtained with a step-like variation of the magnitude of fluctuations (**k**) for *σ*^(0)^ = 0.15 and 0.25, respectively.

Our active model tissues explore the energy landscape at a fixed cell number by rearranging the cells and dissipating the energy through friction with the environment^14^. Because of the complex energy landscape, there is no guarantee that the system reaches the global minimum by the end of simulation. Instead, due to the decreasing active T1 rate *k*_T1_, which gradually drops to zero in each simulation, it is plausible that the final shell morphologies are trapped in local energy minima. If true, this would explain why branched morphologies are only found in simulations that include active cell rearrangements (Fig. 1f). To test this, we first vary the initial active T1 rate ${k}_{{\rm{T}}1}^{(0)}$ at fixed *α* = 0.7 and *β* = 0.5, which give a branched morphology at ${k}_{{\rm{T}}1}^{(0)}=200$. Importantly, we find that this morphology develops only when ${k}_{{\rm{T}}1}^{(0)}$ is high enough and that it has a significantly higher energy than the corresponding minimal-energy shape (Fig. 2a, b). This result shows that the branched epithelial shells are inherently out-of-equilibrium shapes that require a sufficiently high degree of junctional activity to form.

Furthermore, these shapes could well depend on the details of the relaxation protocol because they are trapped in local energy minima. To challenge this possibility, we replace the linear temporal profile of the active T1 rate by a step-like profile, where the initial period with a constant T1 rate ${k}_{{\rm{T}}1}={k}_{{\rm{T}}1}^{(0)}> 0$ of duration *t* = 500 is followed by an equally long period with no T1 transitions (*k*_T1_ = 0). We again find that the branched morphologies only occur if ${k}_{{\rm{T}}1}^{(0)}$ is high enough during the initial period and the final shapes stay qualitatively the same as before (Fig. 2e–g). The branched morphology is formed soon after the beginning of the active phase as illustrated by the sequence of shapes in Supplementary Fig. 7. Thus neither the duration nor the precise temporal profile of the activity are essential for the development of the branches, provided that the duration is long enough.

We further test how the formation of the branched morphology depends on the implementation of junctional activity by considering an entirely different scheme where T1 transitions arise directly from fluctuations of tensions along cell–cell junctions. To this end, we extend the energy function [Eq. (1)] by a line-tension term

4 $${w}_{\gamma }=\mathop{\sum }\limits_{i = 1}^{{\mathcal{E}}}{\gamma }_{i}(t)\left({l}_{{\rm{a}}i}+{l}_{{\rm{b}}i}\right)\ ,$$

describing fluctuations of tensions at cell–cell junctions, i.e., along the direction perpendicular to the apico-basal axis. Here the sum runs over all ${\mathcal{E}}$ cell–cell junctions; *l*_a*i*_ and *l*_b*i*_ are lengths of the apical edge and the basal edge, respectively, that correspond to the *i*th junction, whereas *γ*_*i*_(*t*) is the effective time-dependent line tension associated with these edges. The tension fluctuations are described by the Ornstein–Uhlenbeck process^18,19^ so that

5 $$\frac{{\rm{d}}{\gamma }_{i}(t)}{{\rm{d}}t}=-\frac{1}{\tau }{\gamma }_{i}(t)+{\xi }_{i}(t)\ .$$

Here *τ* is the time scale of the turnover of molecular motors (set to unity, *τ* = 1) and *ξ*_*i*_(*t*) is the Gaussian white noise with properties 〈*ξ*_*i*_(*t*)〉 = 0 and $\langle {\xi }_{i}(t){\xi }_{j}(t^{\prime} )\rangle =(2{\sigma }^{2}/\tau ){\delta }_{i,j}\delta (t-t^{\prime} )$, where *σ*^2^ is the long-time variance of the tension fluctuations. If the magnitude of fluctuations *σ* is sufficiently large, individual edges occasionally shrink to zero length, initiating a T1 transition (see “Methods” section). We analyze two variants of the tension-fluctuation scheme of T1 transitions. In the first one, *σ* decreases linearly from some initial value *σ*^(0)^ to zero (Fig. 2h–j), whereas in the second it has a step-like temporal profile where a period of constant tension fluctuations with *σ* = *σ*^(0)^ is followed by an equally long period with *σ* = 0 (Fig. 2k–m). In agreement with our initial scheme of active noise, branched morphologies are formed in both variants provided that the activity parameter *σ*^(0)^ is large enough (Fig. 2j, m).

To better understand branch formation, we examine the in-plane tissue structure represented by the topology of the polygonal network of cell–cell junctions. We find that the branched shapes obtained in the more active epithelial shells develop very different cell arrangements compared to the less active non-branched shapes (Fig. 2a). In particular, the branched shapes contain many pentagonal and heptagonal cells, which accumulate at the tips of the branches and at their bases, respectively. The distribution of these cells within the shell can be quantified by plotting their pair correlations as functions of the topological distance *d* defined as the integer shortest path between two cells; *d* = 1 for the nearest neighbors, *d* = 2 for the next-nearest neighbors, etc. (Fig. 2c; see “Methods” section). For pentagons, we observe a bimodal distribution with a small peak at *d* = 2, which corresponds to the distance between pentagons within the same branch, and a large peak at *d* = 10 corresponding to the distance between pentagons in different branches. In contrast, the distribution of heptagons, which are mostly located at the bases of the branches, consists of a single peak at *d* ≈ 6. Furthermore, since the distance between the bases is shorter than that between the tips, heptagons are more likely to be found closer to other heptagons than pentagons are to other pentagons from different branches. Note that these pair correlations are qualitatively similar in the budded shapes, which favor pentagons at the buds and heptagons in the saddle-like parts. In contrast, stomatocytes and spheres lack such features and are associated with unimodal distributions (Supplementary Fig. 8).

These results suggest that defects in cell packing, established through active cell rearrangements, may be crucial for shape formation. We hypothesize that the observed distributions of pentagonal and heptagonal cells in the branched morphologies develop hand in hand with the shape itself. In particular, the high-activity conditions enable the formation of a temporarily biased distribution of defects in cell packing, in which pentagons and heptagons act as a seeds for positive and negative Gaussian curvature, respectively (Fig. 2d; Supplementary Movie 3). In turn, once the distribution of the Gaussian curvature is established, pentagons become locked at the positive-Gaussian-curvature tips, whereas heptagons are bound to the saddles. This interplay between the in-plane cell organization and the Gaussian curvature preserves the overall shape of the shell and thus causes it to become trapped away from the global energy minimum. In contrast, shells that are never exposed to a high activity cannot develop the required distribution of pentagons and heptagons, which prevents the formation of multiple localized regions of positive-Gaussian curvature necessary to develop branches.

Surprisingly, even though the relation between the local Gaussian curvature of the surface and locations of the defects in packing of constituents is well known^20^, its relevance for tissue development has not yet been pointed out. This mechanism may well play an important role in cell turnover. Indeed, in one of the fastest renewing organs in mammals—the intestine—cells divide at the bases of the villi and are extruded at the tips^10^. It could be that cell extrusion is initiated by the presence of cells with pentagonal bases. These cells may accumulate at the tips of the villi where their basal side is larger than their apical side simply because pentagonal cells are energetically favorable at positive-Gaussian curvatures (Fig. 2d; Supplementary Fig. 9a). With their truncated-pyramid shape, such cells could more easily delaminate from the tissue. In contrast, cells located at the bases of the villi where the Gaussian curvature is negative are preferentially heptagonal. In these cells, the local saddle-like tissue shape is consistent with the somewhat anisometric shapes of the apical and the basal side, with the two long axes roughly perpendicular to each other (Fig. 2d; Supplementary Fig. 9b–d). As the areas of the two sides should be roughly the same, cells of such a form are stabilized against extrusion and act as anchors. Overall, this hypothesis suggests that the curvature-dependent extrusion rate observed in intestinal epithelia^21^ could also be of mechanical origin. Furthermore, our findings suggest that the basis of this mechanism in epithelial shells may be established spontaneously during shape formation.

### Curvature-thickness coupling

To better understand the obtained shapes, we recast the cell-level energy Eq. (1) in the continuum limit following ref. ^17^. We approximate cell shape by a truncated right square pyramid whose in-plane isometry imposes a fluid-like response to local shear stress. Cell shape is parametrized by local principal curvatures *c*_1_ and *c*_2_ of the midplane as well as by tissue thickness *h*, i.e., cell height, measured in units of $1/{V}_{{\rm{cell}}}^{1/3}$ and ${V}_{{\rm{cell}}}^{1/3}$, respectively (Supplementary Note 1). The dimensionless harmonic elastic energy per unit area reads

6 $$\frac{{\rm{d}}w}{{\rm{d}}a}	= \, (\alpha +\beta )+2\left[1-\frac{{\left(\alpha -\beta \right)}^{2}}{4}\right]\sqrt{{h}^{3}}+\frac{\sqrt{h}}{8}{\left[{c}_{1}+{c}_{2}-2\sqrt{h}(\alpha -\beta )\right]}^{2}\\ 	\,\,\,\,\,\,\,+\left(\frac{\alpha +\beta }{4}+\frac{\sqrt{{h}^{3}}}{12}-\frac{1}{4\sqrt{{h}^{3}}}\right){h}^{2}{c}_{1}{c}_{2}.$$

The first two terms together represent the surface tension, whereas the third and the fourth term are the local and the Gaussian bending energy per unit area, respectively. Note that the thickness *h* in Eq. (6) is an independent variable. While it is constant in a flat tissue at *α* = *β*, in the nontrivial shapes *h* is coupled to the local curvature. As a result, the surface tension varies along the surface and so do the local bending modulus $\sqrt{h}/8$, the spontaneous curvature ${c}_{0}=2\sqrt{h}(\alpha -\beta )$, and the Gaussian modulus [i.e., the last term in Eq. (6) divided by the Gaussian curvature *c*_1_*c*_2_]. These features render the tissue energy functional quite different from the usual surface and bending energies of solid or fluid membranes.

**Fig. 3:**
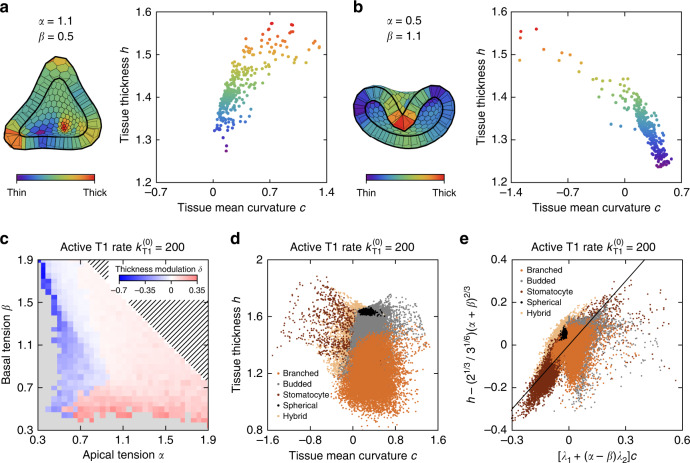
**Thickness-curvature coupling arises due to apico-basal polarity**. **a**, **b** Thickness-curvature portraits of the active T1 (${k}_{{\rm{T}}1}^{(0)}=200$) budded shape with *α* = 1.1, *β* = 0.5 (**a**) and of a stomatocyte shape with *α* = 0.5, *β* = 1.1 (**b**); each cell is represented by a point. In the cutaway view of the model epithelial shells, tissue thickness is represented using color code, which is also used for the points in the two diagrams. **c** Modulation of tissue thickness *δ* in shapes in the (*α*, *β*)-plane for ${k}_{{\rm{T}}1}^{(0)}=200$. The gray region represents the non-physical regime, whereas the hatched region denotes the spherical shapes at *α* + *β* > 2.6. **d** The 56,400 cell-by-cell *h*(*c*) datapoints from the 188 model shapes from panel c with *α* + *β* < 1.9. **e** Datapoints from panel **d** collapsed by rescaling described in the text; also plotted is the line of identity.

The coupling between the mean curvature and the thickness (*β* − *α*)(*c*_1_ + *c*_2_)*h*/2, contained in the local bending term, is the most important of the above effects. In the more elaborate shapes, this coupling is quite pronounced as illustrated by Fig. 3a, b showing the thickness-curvature portraits of a budded and a stomatocyte shape, respectively. In these two diagrams, each cell is represented by a point indicating the mean curvature *c* = (*c*_1_ + *c*_2_)/2 and thickness *h* of the tissue at this cell (see “Methods” section). The positive correlation between thickness and curvature is evident in the budded shape (Fig. 3a) as is the negative correlation in the stomatocyte (Fig. 3b). In the budded *α* = 1.1, *β* = 0.5 shape with a positive spontaneous curvature *c*_0_ (i.e., a spontaneous curvature with the same sign as the curvature of a sphere), the taller cells are located at the buds where the mean curvature is largest (Fig. 3a), whereas in the *α* = 0.5, *β* = 1.1 stomatocyte where *c*_0_ < 0, they are concentrated at the invagination (Fig. 3b).

To quantify the curvature-thickness coupling throughout the phase diagram, we introduce the relative tissue thickness modulation $\delta =p\cdot \Delta h/\overline{h}$, where Δ*h* is the amplitude of thickness modulation, whereas $\overline{h}$ is the average cell height in the epithelial shell; here *p* = +1 if curvature and thickness are correlated (Fig. 3a) and *p* = −1 if they are anticorrelated (Fig. 3b). We find that the thickness modulation is sizable in all non-spherical shapes, with ∣*δ*∣ reaching about 0.65 in stomatocytes and 0.35 in budded and branched shapes (Fig. 3c). Consistent with the continuum theory [Eq. (6)], the sign of *δ* agrees with the sign of the modulus of the curvature-thickness coupling *α* − *β*.

We can further analyze the coupling between curvature and thickness by fitting the *h*(*c*) plots for individual epithelial shells by linear functions. We then plot the vertical intercept *n* of these functions against tissue tension *α* + *β* and the slopes *k* against the differential tension *α* − *β* for all 188 model shapes discussed (Supplementary Fig. 10). The vertical intercept *n* can be described by the above analytical solution for hexagonal cells in a flat epithelium $n\approx \left({2}^{1/3}/{3}^{1/6}\right){(\alpha +\beta )}^{2/3}$, whereas the slope *k* can be approximated by *λ*_1_ + (*α* − *β*)*λ*_2_, where *λ*_1_ = −0.059 ± 0.004 and *λ*_2_ = 0.45 ± 0.01 are fitting parameters. (These two observations only apply to shells with *α* + *β* < 1.9. Beyond this threshold, our epithelial shells approach a spherical shape and their midplane area is defined by the enclosed volume rather than by *α* and *β*.) We can then rescale $h\to h-\left({2}^{1/3}/{3}^{1/6}\right){(\alpha +\beta )}^{2/3}$ and $c\to \left[{\lambda }_{1}+(\alpha -\beta ){\lambda }_{2}\right]c$, which allows us to collapse all *h*(*c*) scatter plots for shells at ${k}_{{\rm{T}}1}^{(0)}=200$ with *α* + *β* < 1.9 (Fig. 3d, e); the same can also be done for shells at ${k}_{{\rm{T}}1}^{(0)}=0$ in Fig. 1e.

While the modulation of cell height is often attributed to cell differentiation^22,23^, our model shows that it can appear even in tissues of identical cells where it is a telltale sign of mechanical apico-basal polarity. Similar shape features are observed in the folded morphologies in a variety of tissues in both vertebrates and invertebrates^13^.

## Discussion

**Fig. 4:**
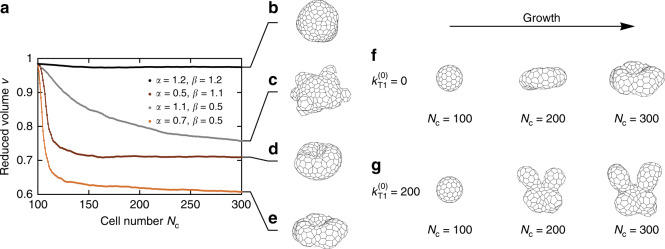
**Model of growing epithelial shells**. **a** Reduced volume of growing shells as a function of cell number, based on 25 instances. **b**–**e** Shell shapes at *N*_c_ = 300 and (*α*, *β*) = (1.2, 1.2), (1.1, 0.5), (0.5, 1.1),  and (0.7, 0.5), respectively. **f** Growing shell with (*α*, *β*) = (0.7, 0.5) at 100-, 200-, and 300-cell stage in absence of active T1 transitions. **g** Growing shell at the same *α* and *β* but with active T1 transitions.

Although our model yields a diverse catalog of morphologies of epithelial shells, their formation in real systems may rely on processes other than surface tension and active cell rearrangements since the shape usually develops while cells grow and divide. To study the effects of growth, we simulate shells at fixed cell-growth and cell-division rates (see “Methods” section) with *τ*_d_ = 2000 being the expected time until a cell next divides, whereas the lumen volume follows Eq. (7) (see “Methods” section). Starting from a sphere of 100 cells, we monitor the growing shells until the number of cells reaches 300. We first compute the shapes in absence of activity (i.e, ${k}_{{\rm{T}}1}^{(0)}=0$) and find that in terms of their final reduced volumes, they agree well with those from our original model (Fig. 4a; Supplementary Fig. 5d–g). The spherical, budded, and stomatocyte shapes all develop their characteristic features, although they are less regular than in the non-growing shells due to a different relaxation mechanism (Fig. 4b–d; Supplementary Movie 4). In contrast, the *α* = 0.7, *β* = 0.5 shape does not develop branches although its reduced volume is similar to that of the branched shapes obtained in active shells at a fixed cell number (Fig. 4a, e, f).

To check whether branching can be recovered in growing epithelial shells by including junctional activity, we combined cell division with the previously used active T1 transitions with a linearly decreasing T1 rate [${k}_{{\rm{T}}1}(t)={k}_{{\rm{T}}1}^{(0)}({t}_{\max }-t)/{t}_{\max }$, where ${k}_{{\rm{T}}1}^{(0)}=200$ and ${t}_{\max }=1000$; this set of parameters causes the active T1 rate to reach zero at a time when the shell contains about 160 cells]. As shown by Fig. 4g and Supplementary Movie 5, this level and duration of active T1 transitions are sufficient for the branched morphology to develop, which agrees with our observation that epithelial shells require a certain degree of junctional activity to branch (Fig. 2a, b).

Our results show that small epithelial shells can form a variety of shapes relying on collective mechanics of non-differentiated cells, coupled through a fluid-like interior. We explore a scenario where a complex shape forms due to a mismatch between the preferred surface area of the shell and the volume of the enclosed lumen. The four characteristic morphologies—spherical, stomatocyte, budded, and branched—are distinguished by the reduced volume. We also develop a continuum theory and use it to explain the fine features of the shapes (specifically, the cell-height modulation) which could be measured in vitro to estimate the apico-basal surface tension polarity. Overall, our model provides clues for the control of organoid shapes in experiments. This can be done by changing the relative apical and basal tensions of cells, which should depend on the biochemical composition of the lumen and the medium. These tensions are best viewed as effective concepts encompassing all membrane-related effects and processes that shape the cells, and may be altered in several ways including, e.g., the activation of the complex cascade leading to contraction of the apical network^24^. Our results also suggest that the organoid shape may be affected by suppression or promotion of junctional activity and by the effect of this activity on the topology of the tissue. A systematic assessment of the different experimental options could conceivably expand the toolbox of the organoid technology and tissue engineering^25^.

A natural extension of this work would be to include planar cell polarity, which can induce tubulogenesis and thus branching^26^, and cell differentiation, which could contribute to self-organization of cells within organoids through differential adhesion as well as to programmed shape formation through spatial modulation of apico-basal polarity. To better describe the typical experimental setup in growing organoids, the model could consider tissue growth on curved substrates and include viscous dissipation at the tissue-matrix interface, which may promote buckling and branching^27^. In such a case, other cell-scale active processes, e.g., cell motility, could contribute to the collective cell dynamics and organoid morphogenesis.

## Methods

### Implementation

Simulations of epithelial-shell shapes are performed within vertX3D, a package of C++ routines that implement the 3D vertex model of epithelial tissues. The initial configuration of cells is a spherical shell enclosing the lumen volume *v*_lumen_. These configurations are constructed by finding the dual of the convex hull to the minimal-energy solution of the Thomson problem on a sphere^28^. The dual determines the positions of the apical vertices, whereas the positions of the basal vertices are calculated by displacing their apical counterparts radially by ${[3({v}_{{\rm{lumen}}}+{N}_{{\rm{c}}})/(4\pi )]}^{1/3}-{[3{v}_{{\rm{lumen}}}/(4\pi )]}^{1/3}$. To correct the initial cell volumes, which do not agree exactly with the preferred volumes, the shells are first relaxed at *k*_T1_ = 0 for a short time before the active T1 rate is set to ${k}_{{\rm{T}}1}^{(0)}$. The preferred cell volumes and the lumen volume are enforced by auxiliary harmonic terms in the energy; the modulus of these terms is very large (100 unless stated otherwise).

In the active T1 scheme, the T1 transitions are initiated as described in the main text; in the fluctuating tension scheme, they are only initiated if the length of a junction expressed in units of ${V}_{{\rm{cell}}}^{1/3}$ falls under 0.01. In both cases, T1 transitions are applied by changing the connectivity of the apical network, and then updating the basal network and the lateral sides accordingly. During the transition, the two apical vertices defining the edge that undergoes the T1 event are first moved to the average of their previous positions. The four-way-rosette arrangement is left for a short time interval of 2 × 10^−3^ before it is resolved. The resolution happens by separating the new pair of apical vertices by a distance 0.0005 in a symmetric fashion.

In epithelial shells with no active T1 transitions (${k}_{{\rm{T}}1}^{(0)}=0$) simulations are run until *t* = 2000, i.e., twice as long as in shells with junctional activity; this is because the stomatocyte shapes at *α* ≈ *β* require a longer time to fully develop.

In our vertex model, steric repulsion between cells is not implemented and self-overlapping shapes are therefore possible. During post-processing, we check each obtained final shape and discard all that self-overlap; temporary overlaps may occur during the simulation.

When analyzing the obtained epithelial-shell shapes, we compute cell height (i.e., tissue thickness) reported in Fig. 3 as the distance between the centroids of the apical and the basal side, and we approximate the mean curvature of each cell by that of a truncated cone with the same apical area, basal area, and height.

When considering growing epithelial shells, cells that are otherwise subject to the fixed-volume constraint enter the growth period with a constant probability d*P*/d*t* = 1/*τ*_d_, where *τ*_d_ = 2000 is the characteristic time scale of division events. During the growth period, the cell volume is increased linearly in time according to d*V*/d*t* = 1/*τ*_g_, where *τ*_g_ = 1 is the cell growth time scale. As soon as the volume is doubled, the cell is divided with the mitotic plane connecting a random pair of opposing lateral sides. Starting with a spherical shell of 100 cells, we let the cells divide while increasing the preferred lumen volume according to Eq. (7), substituting *N*_c_ by the total volume of the tissue.

### Volume and active T1 rate rescaling

When comparing morphologies with different cell numbers *N*_c_ in Supplementary Fig. 3, the active T1 rate *k*_T1_ and lumen volume *v*_lumen_ must be appropriately rescaled for the comparison to be relevant. As the probability for an individual edge to undergo a T1 transition is ${k}_{{\rm{T}}1}\delta t/{\mathcal{E}}$, the most relevant comparison is between model epithelial shells where an individual cell–cell junction has the same probability of undergoing a T1 at both *N*_c_ in question. We therefore rescale ${k}_{{\rm{T}}1}\to {k}_{{\rm{T}}1}{{\mathcal{E}}}_{{N}_{{\rm{c}}}}/{{\mathcal{E}}}_{300}$; here ${{\mathcal{E}}}_{{N}_{{\rm{c}}}}$ is the number of cell–cell junctions in an shell of *N*_c_ cells and ${{\mathcal{E}}}_{300}$ is their number in a 300-cell shell. The same rescaling is also used when considering growing epithelial shells (Fig. 4) so that edges have an equal probability of undergoing an active T1 transition regardless of the number of cells.

When rescaling lumen volumes, we choose them such that the model epithelial shells have the same *α*, *β* threshold for the spherical shape, which can be estimated as follows. As before, we approximate cell height by ${h}_{0}=\left({2}^{1/3}/{3}^{1/6}\right){(\alpha +\beta )}^{2/3}$. The threshold for a spherical shape is then the value of tissue tension *α* + *β* at which a spherical shell of thickness *h*_0_ around the lumen of volume *v*_lumen_(*N*_c_) has volume *N*_c_. We then equate the *α* + *β* values at the given target *N*_c_ and the reference *N*_c_ = 300, giving the condition

7 $${\left[{v}_{{\rm{lumen}}}({N}_{{\rm{c}}})+{N}_{{\rm{c}}}\right]}^{1/3}-{\left[{v}_{{\rm{lumen}}}({N}_{{\rm{c}}})\right]}^{1/3}={\left[{v}_{{\rm{lumen}}}({N}_{{\rm{c}}})+300\right]}^{1/3}-{\left[{v}_{{\rm{lumen}}}(300)\right]}^{1/3}.$$

Here *v*_lumen_(300) = 100 is the lumen volume at 300 cells, whereas *v*_lumen_(*N*_c_) is the lumen volume at the target cell number *N*_c_. This equation can be solved for *v*_lumen_(*N*_c_).

### Topological pair correlations

The topological pair correlation function *g*_*γ*_(*d*) encodes how many *γ*-sided cells may on average be expected at a topological distance *d* from another *γ*-sided cell. It is defined as

8 $${g}_{\gamma }(d)=\left\langle \frac{1}{{n}_{\gamma }}\mathop{\sum }\limits_{i = 1}^{{n}_{\gamma }}{{\mathcal{N}}}_{\gamma i}(d)\right\rangle .$$

Here the sum runs over all *γ*-sided cells and ${{\mathcal{N}}}_{\gamma i}(d)$ is the number of *γ*-sided cells at a topological distance *d* from the *i*th *γ*-sided cell. The normalizing prefactor *n*_*γ*_ is the total number of *γ*-sided cells in the shell. The expression in the brackets is averaged over 300 simulation runs.

### Shape anisometry

The long axes of the apical and basal sides of cells in Fig. 2d and Supplementary Fig. 9 are obtained by diagonalizing the gyration tensor computed from the vertices of a side. The anisometry factor of a cell side *κ* is given by *κ* = (*g*_1_ − *g*_2_)/(*g*_1_ + *g*_2_), where *g*_1_ and *g*_2_ are the largest and the second largest eigenvalue of the gyration tensor, respectively; as cell sides are slightly non-planar, the third eigenvalue is finite rather than 0 but still much smaller than *g*_1_ and *g*_2_.

### Reporting summary

Further information on research design is available in the Nature Research Reporting Summary linked to this article.

## Supplementary information

Supplementary Information

Supplementary Movie 1

Supplementary Movie 2

Supplementary Movie 3

Supplementary Movie 4

Supplementary Movie 5

Description of Additional Supplementary Files

Reporting Summary

## Data Availability

Data supporting the findings of this manuscript are available from the corresponding author upon reasonable request. A reporting summary for this Article is available as a Supplementary Information file.
