# Supplementary material for: Collective cell mechanics of epithelial shells with organoid-like morphologies
Source: Nat Commun. 2020 Jul 30;11:3805. doi: 10.1038/s41467-020-17535-4 (PMC7393134; doi:10.1038/s41467-020-17535-4)
Supplement: Supplementary file 1 — Supplementary Information [file 41467_2020_17535_MOESM1_ESM.pdf]

Supplementary Information

Collective cell mechanics of epithelial shells with organoid-like morphologies

Rozman et al.

Supplementary Figures

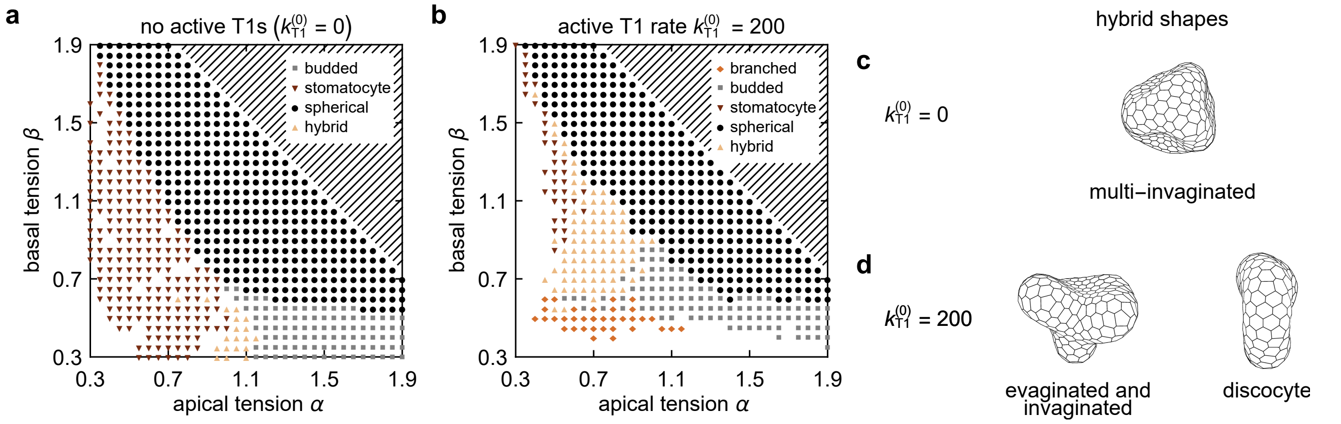

Supplementary Figure 1. Morphological phase diagrams of epithelial shells. a Raw data at $k_{\rm T1}^{(0)} = 0$. Shapes with $\alpha + \beta > 2.6$ (hatched region) are spherical and not shown for clarity. Self-intersecting and otherwise non-physical shapes at small α or β are not included (Methods). **b** Raw data at $k_{T1}^{(0)} = 200$. The hatched region again denotes spherical shapes with $\alpha + \beta > 2.6$, and self-intersecting and otherwise non-physical shapes are not shown. **c** Example of a hybrid shape with multiple invaginations at $N_{\rm c} = 100$, $v_{\rm lumen} = 100$, and $k_{\rm T1}^{(0)} = 0$, with $\alpha = 1.1$ and $\beta = 0.4$. **d** Examples of hybrid shapes at the same $N_{\rm c}$ and $v_{\rm lumen}$, but with $k_{\rm T1}^{(0)} = 200$. Here hybrid shapes appear at $\alpha \approx \beta$ and often feature either both evaginations and invaginations (left; $\alpha = 0.7$ and $\beta = 0.65$) or multiple invaginations, giving rise to a discocyte-like shape (right; at $\alpha = 0.55$ and $\beta = 0.7$).

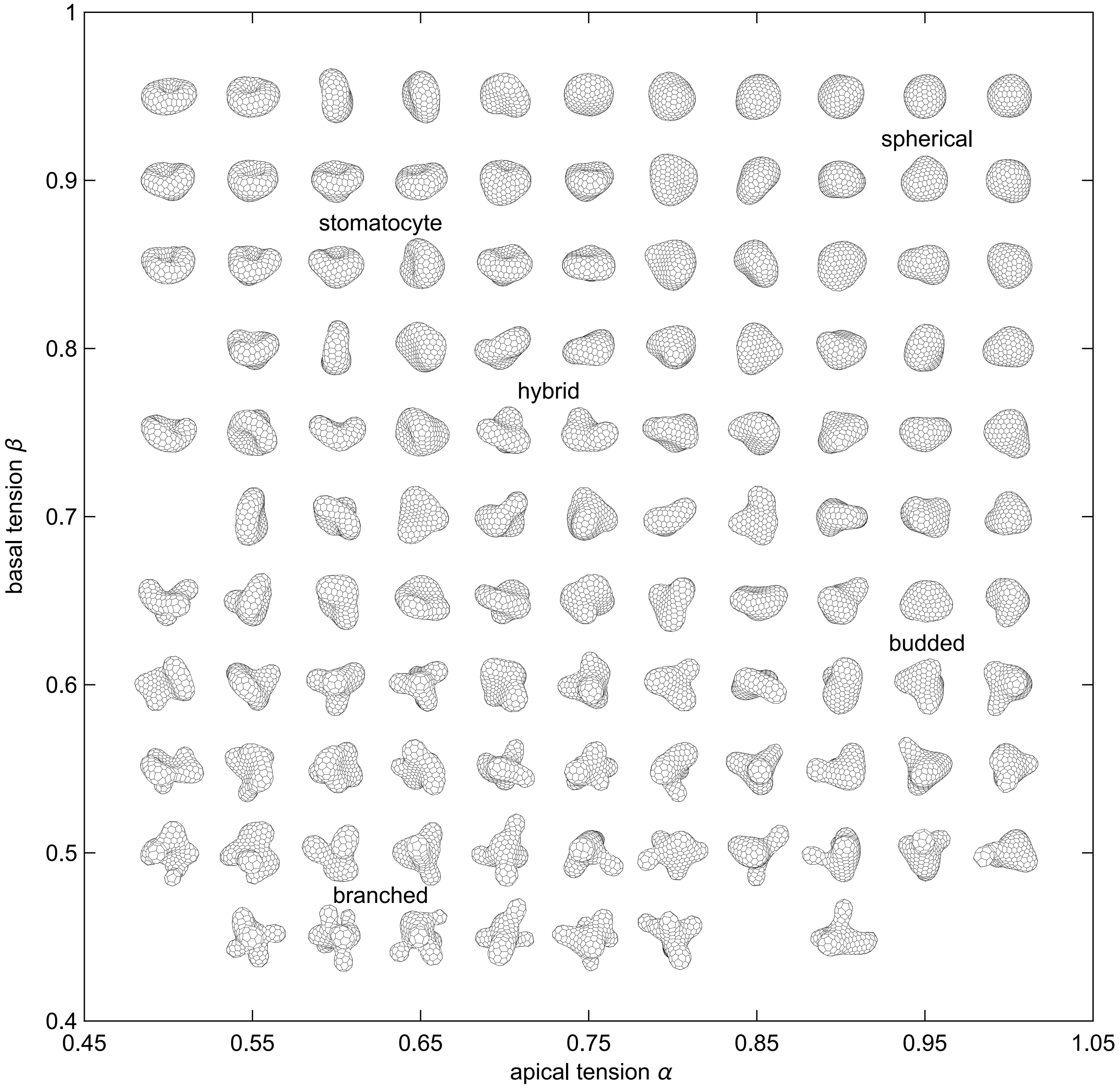

Supplementary Figure 2. Diagram of epithelial-shell shapes. Shapes from the bottom-left region of the phase diagram in Fig. 1f of the main text with $k_{\mathrm{T1}}^{(0)} = 200$, showing various budded, branched, stomatocyte, and hybrid shapes as well as some spherical shapes.

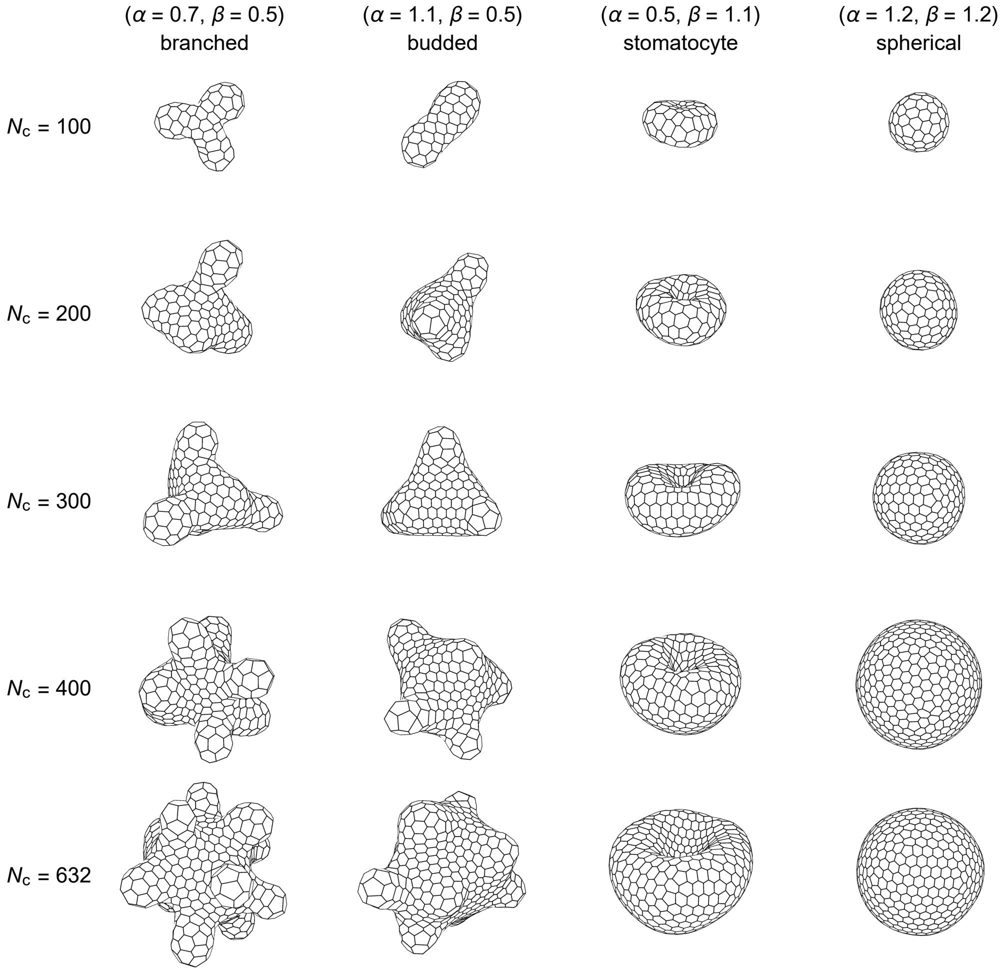

Supplementary Figure 3. Comparison of epithelial shells containing different numbers of cells. Shell morphologies at $k_{\mathrm{T1}}^{(0)} = 200 \times \mathcal{E}_{N_\mathrm{c}}/\mathcal{E}_{300}$ for $N_\mathrm{c} = 100, 200, 300, 400$, and 632 at parameters ($\alpha = 0.7$, $\beta = 0.5$), ($\alpha = 1.1$, $\beta = 0.5$), ($\alpha = 0.5$, $\beta = 1.1$), and ($\alpha = 1.2$, $\beta = 1.2$); $\mathcal{E}_{N_\mathrm{c}}$ is the number of cell-cell junctions of a shape at a given number of cells N_c (Methods). Given an appropriate rescaling of lumen volumes, the same model parameters give the same type of morphology.

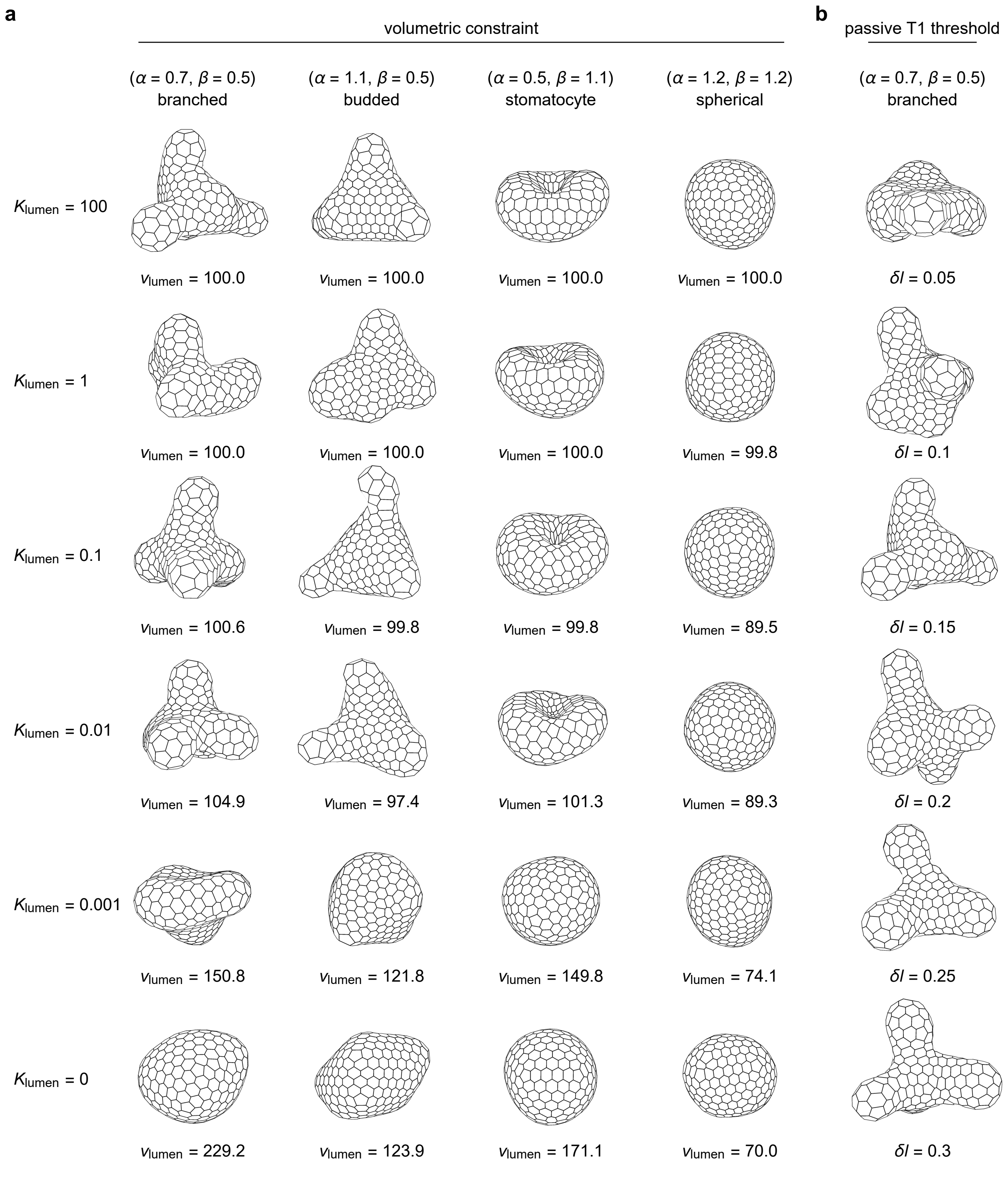

Supplementary Figure 4. Influence of modulus of lumen volumetric term and passive T1 transition threshold length of shell morphologies. a Shell morphologies at moduli of the auxiliary lumen volumetric term K_{lumen} equal to $100, 1, 0.1, 0.01, 0.001$ for four sets of α and β. The preferred lumen volume equals 100 in dimensionless units; the actual lumen volume is given under each shape. If K_{lumen} is too small, the epithelial shells are devoid of clear morphological features. **b** Shell morphologies at $\alpha = 0.7$, $\beta = 0.5$ at different values of the threshold for passive T1 transitions δl. Branches only develop at sufficiently large δl.

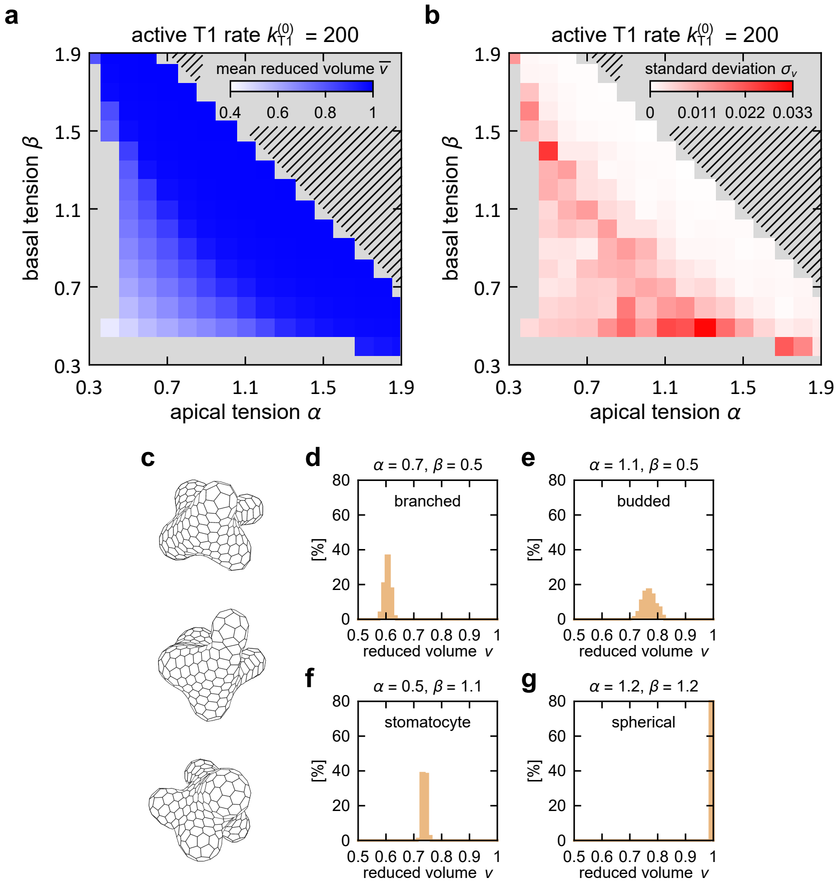

Supplementary Figure 5. Analysis of reduced volume of active epithelial shells. **a** Arithmetic mean of the reduced volume from 10 computed shells. The reduced volume of a shape depends almost exclusively on the tissue tension $\alpha + \beta$ and is virtually independent of the differential tension $\alpha - \beta$. **b** Standard deviation of the reduced volumes from the 10 simulation runs, showing that the reduced volume of the shape at a given α and β is well-defined. **c** Three instances of a branched shell at $\alpha = 0.7$, $\beta = 0.5$, $N_c = 300$, $v_{\rm lumen} = 100$, and $k_{\rm T1}^{(0)} = 200$. **d-g** Distributions of reduced volumes for 300 instances of shapes at α and β corresponding to the branched, budded, stomatocyte, and spherical morphologies in Fig. 1d of the main text.

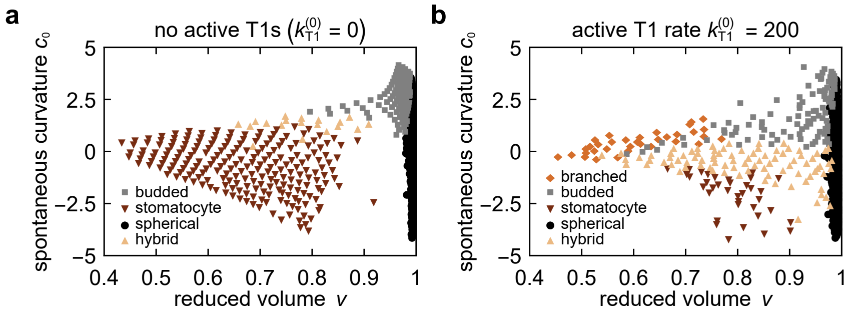

Supplementary Figure 6. Comparison between epithelial shells and lipid vesicles. a Phase diagram of epithelial shells with no active T1 transitions ($k_{\mathrm{T1}}^{(0)} = 0$) from Supplementary Fig. 1a plotted in the space spanned by the reduced volume v [Eq. (2) of the main text] and spontaneous curvature $c_0 = 2(\alpha+\beta)^{1/3}(\alpha-\beta)$ [Eq. (22) of Supplementary Note 1]. **b** Phase diagram of $k_{\mathrm{T1}}^{(0)} = 200$ epithelial shells from Supplementary Fig. 1b plotted in the (v, c_0)-plane. The trumpet-like arrangement of points in panel b follows from the fact that in any stable shape, both α and β are restricted in magnitude: If one of them (say α) is negative, its absolute value should not exceed a certain threshold or else the energy of individual cells is unbounded from below. By considering completely flattened squamous cells, we find that $|\alpha| < \min(1/2, \beta)$, which in turn imposes a restriction on the magnitude of c_0. In the numerically obtained closed shell shapes, the actual restrictions are somewhat different from those obtained by analyzing the shape of individual cells but the qualitative form of the physically relevant domain is still trumpet-like although not uniformly populated. In panel a, the trumpet form of the domain is primarily due to the mapping of the data from Supplementary Fig. 1a; most $k_{\mathrm{T1}}^{(0)} = 0$ shapes are unaffected by the above restrictions, which are less limiting than in their generally more elaborated $k_{\mathrm{T1}}^{(0)} = 200$ counterparts. The locations of the different morphologies in panels a and b are reasonably similar to those of their vesicle analogs as seen in Fig. 10 of Ref. [1].

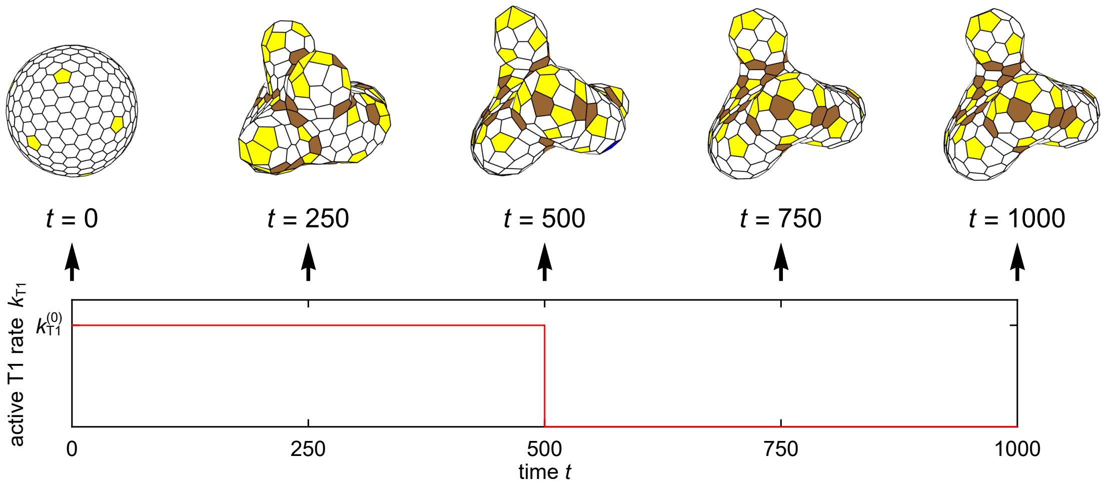

Supplementary Figure 7. Sequence of shapes during step-like active T1 transitions scheme. Shapes of $\alpha = 0.7, \beta = 0.5$ model epithelial shells at different stages of the step-like protocol. While the initial $t = 0$ shape is spherical, the $t = 250$ shape shows that branches are formed rather quickly during the active period. The similarity of the $t = 250$ and the $t = 500$ shapes suggests that the duration of the active period is not very important as long as it is long enough, and that further extending it would not affect the shapes very much.

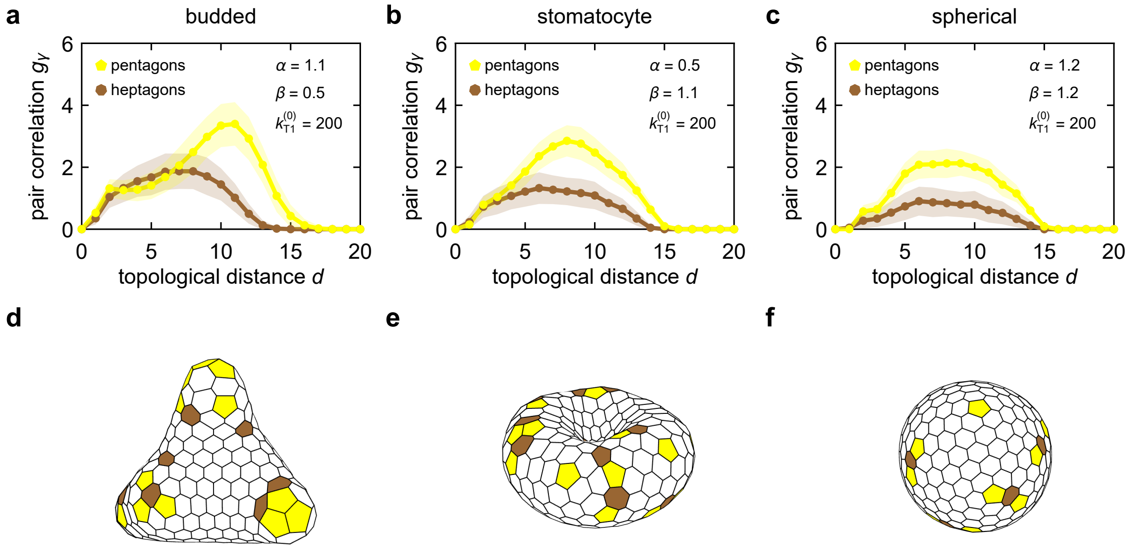

Supplementary Figure 8. Topology-geometry coupling is also seen in budded morphologies, but is absent in stomatocyte and spherical epithelial shells. Topological pair correlation function $g_\gamma(d)$ of pentagonal and heptagonal cells in budded, stomatocyte, and spherical shapes (**a**, **b**, and **c**, respectively, with model parameters given in the legend). Solid lines are guides to the eye through arithmetic means over 300 instances of shapes shown by symbols, whereas shaded areas show the standard deviation. The correlation function in the budded shapes is similar to that in the branched shapes (Fig. 2c of the main text), but its features are less pronounced. In the stomatocytes and in the spherical shapes, the form of g_γ approaches $\sin d$ expected in a random distribution of points on a sphere, with little or no difference in the topological distance corresponding to the maximal value for pentagons and heptagons. Note that the magnitudes of g_5 and g_7 are different because the total number of pentagons n_5 exceeds the total number of heptagons n_7 by 12 for topological reasons. (**d-f**) Representative shapes for the set of model parameters corresponding to each type of epithelial shells.

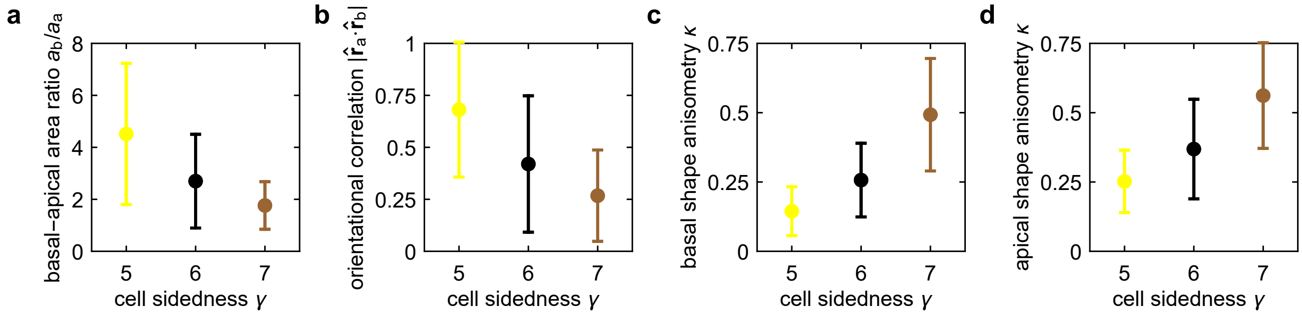

Supplementary Figure 9. Characteristics of cell shape in branched shells depend significantly of cell sidedness. a Ratio of the area of the basal and the apical cell side in pentagonal, hexagonal and heptagonal cells for the model epithelial shell in Fig. 2d of the main text [$\alpha = 0.7, \beta = 0.5, k_{\mathrm{T1}}^{(0)} = 200$]. **b** Orientational correlation between the long axes of the apical and basal cell sides ($\hat{\mathbf{r}}_\mathrm{a}$ and $\hat{\mathbf{r}}_\mathrm{b}$, respectively; Methods) for the same shell, computed as the magnitude of the dot product of unit vectors pointing along the two long axes. **c** and **d** Shape anisometry κ of basal and apical cells, respectively, in the epithelial shell in Fig. 2d of the main text, with larger values corresponding to more anisometric shapes (Methods). In all panels, symbols represent the arithmetic mean whereas the error bars denote the standard deviation.

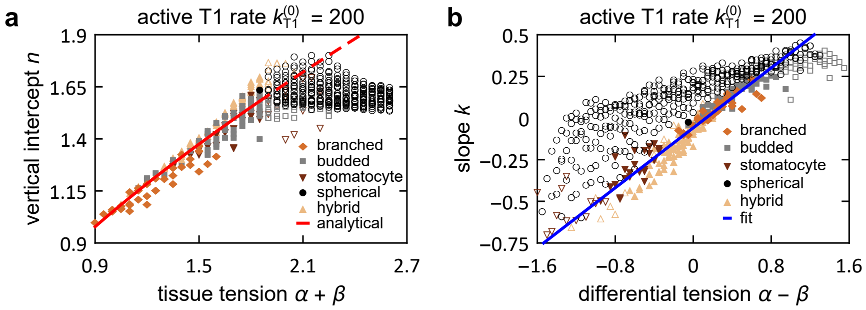

Supplementary Figure 10. Analysis of curvature-thickness coupling. **a** Vertical intercepts as a function of tissue tension $\alpha+\beta$ obtained by fitting the $h(c)$ plot by linear functions for all epithelial shells in Fig. 1f of the main text. Points with $\alpha+\beta \geq 1.9$ where the shells approach a sphere are denoted by open symbols. The red line shows the function $\left(2^{1/3}/3^{1/6}\right)(\alpha+\beta)^{2/3}$, its dashed section corresponding to $\alpha+\beta \geq 1.9$. **b** Slopes k for the same epithelial shells as a function of differential tension $\alpha-\beta$. Open symbols are again for points with $\alpha+\beta \geq 1.9$. Blue line shows the linear fit of the type $\lambda_1 + (\alpha-\beta)\lambda_2$.

Supplementary Note 1

3D continuum theory

The derivation of the continuum theory is based on generalizing the approach reported in Ref. [2] to three dimensions. We begin by defining a model cell represented by a truncated pyramid with a rectangular base and a volume of V_{cell}. The model cell is defined by the principal curvatures C_1 and C_2, height H, and the aspect ratio in the midplane located halfway between the apical and the basal side defined by

$$\nu = \frac{\Delta s_1}{\Delta s_2}, \tag{1}$$

where Δs_1 and Δs_2 are the length and the width of the rectangular midplane cross section, respectively (Supplementary Fig. 11). These four parameters can describe four deformation modes: (i) and (ii) bending in the directions of the principal curvatures, (iii) changes in the local tissue thickness, and (iv) pure shear deformation.

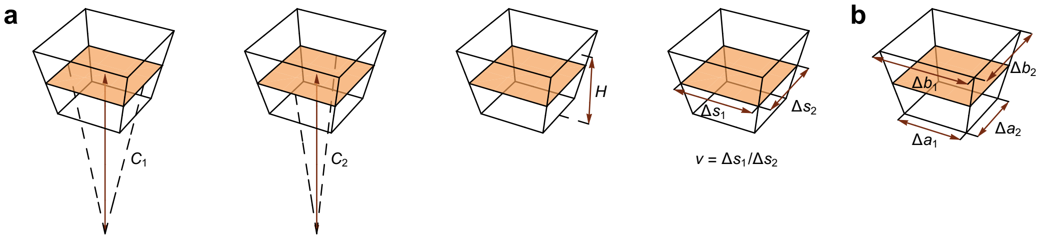

Supplementary Figure 11. Parametrization of model cell. a The model cell represented as a truncated pyramid of volume V_{cell} is defined by the principal curvatures C_1 and C_2, height H, and aspect ratio of the midplane ν; the orange rectangle represents the midplane. **b** Lengths of edges of the apical and the basal side which depend on C_1, C_2, H, and ν.

The geometrical parameters of the model cell can be related to the length and the width of the apical side Δa_1 and Δa_2, respectively, and the length and the width of the basal sides Δb_1 and Δb_2, respectively, by

$$\Delta a_1 = \frac{1 - C_1 H/2}{1 + C_1 H/2} \Delta b_1 \tag{2}$$

and

$$\Delta a_2 = \frac{1 - C_2 H/2}{1 + C_2 H/2} \Delta b_2. \tag{3}$$

Upon inserting the relations $\Delta s_1 = (\Delta a_1 + \Delta b_1)/2$ and $\Delta s_2 = (\Delta a_2 + \Delta b_2)/2$ into Eq. (1) and using Eqs. (2) and (3), we obtain

$$\Delta b_2 = \frac{1}{\nu} \frac{1 + C_2 H/2}{1 + C_1 H/2} \Delta b_1. \tag{4}$$

Lastly, by combining the expression for the midplane area $\Delta A_{\text{m}} = \Delta s_1 \Delta s_2$ with the above relations, we find that

$$\Delta b_1^2 = \nu \left(1 + C_1 H/2\right)^2 \Delta A_{\text{m}}. \tag{5}$$

Equations (2)-(5) allow us to express the lengths of edges of the apical and basal sides in terms of C_1, C_2, ν, H, and ΔA_{m}.

We now wish to derive the elastic energy per unit area. For a section of tissue with midplane area ΔA_{m} at fixed C_1, C_2, ν, and H, the energy reads

$$\Delta W = \Gamma_{\text{a}} \Delta A_{\text{a}} + \Gamma_{\text{b}} \Delta A_{\text{b}} + \frac{\text{d}W_{\text{l}}}{\text{d}A_{\text{m}}} \Delta A_{\text{m}}\,, \tag{6}$$

where Γ_{a}, Γ_{b}, and Γ_{l} are the apical, basal and lateral surface tensions, respectively, ΔA_{a} and ΔA_{b} are the apical and basal areas corresponding to ΔA_{m}, whereas $\text{d}W_{\text{l}}/\text{d}A_{\text{m}}$ is the energy density associated with the lateral sides.

The energy terms associated with the apical and the basal sides can be rewritten by using $\Delta A_{\rm a} = \Delta a_1 \Delta a_2$, and $\Delta A_{\rm b} = \Delta b_1 \Delta b_2$ along with Eqs. (2)-(5) such that

$$\Gamma_{\rm a}\Delta A_{\rm a} + \Gamma_{\rm b}\Delta A_{\rm b} = (\Gamma_{\rm a}+\Gamma_{\rm b})\left(1+\frac{C_1 C_2 H^2}{4}\right)\Delta A_{\rm m} + (\Gamma_{\rm b}-\Gamma_{\rm a})\frac{(C_1+C_2)H}{2}\Delta A_{\rm m}. \tag{7}$$

The derivation of the term associated with the lateral sides is more involved. We first calculate the energy of the lateral sides of a single cell with a volume of $V_{\rm cell}$ and fixed C_1, C_2, ν, and H. This energy reads

$$W_{\rm l} = \frac{1}{2}\Gamma_{\rm l}(2A_{\rm l1} + 2A_{\rm l2}), \tag{8}$$

where $A_{\rm l1}$ is the area of the lateral side with edges Δa_1 and Δb_1 whereas $A_{\rm l2}$ is the area of the lateral side with edges Δa_2 and Δb_2. By using simple trigonometry and relations derived above, $A_{\rm l1}$ and $A_{\rm l2}$ can be recast as

$$A_{\rm l1} = \frac{1}{1+C_1H/2}\Delta b_1\sqrt{H^2 + \left(\frac{C_2H/2}{1+C_1H/2}\right)^2\frac{1}{\nu^2}\Delta b_1^2} \tag{9}$$

and

$$A_{\rm l2} = \frac{1}{\nu}\frac{1}{1+C_1H/2}\Delta b_1\sqrt{H^2 + \left(\frac{C_1H/2}{1+C_1H/2}\right)^2\Delta b_1^2}, \tag{10}$$

respectively. Now we combine these results with the expression for the volume of a single cell with given Δa_1, Δa_2, Δb_1, Δb_2, and H, which reads

$$\begin{aligned} V_{\rm cell} &= \left(\Delta a_1\Delta a_2 + \frac{1}{2}\Delta a_1\Delta b_2 + \frac{1}{2}\Delta b_1\Delta a_2\Delta b_1\Delta b_2\right)\frac{H}{3} \\ &= \frac{1+C_1C_2H^2/12}{(1+C_1H/2)^2}\frac{1}{\nu}\Delta b_1^2 H, \end{aligned} \tag{11}$$

and we obtain

$$A_{\rm l1} = \sqrt{\nu}\frac{1}{\sqrt{1+C_1C_2H^2/12}}\sqrt{\frac{V_{\rm cell}}{H}}\sqrt{H^2 + \frac{1}{\nu}\frac{(C_2H/2)^2}{1+C_1C_2H^2/12}\frac{V_{\rm cell}}{H}} \tag{12}$$

and

$$A_{\rm l2} = \frac{1}{\sqrt{\nu}}\frac{1}{\sqrt{1+C_1C_2H^2/12}}\sqrt{\frac{V_{\rm cell}}{H}}\sqrt{H^2 + \nu\frac{(C_1H/2)^2}{1+C_1C_2H^2/12}\frac{V_{\rm cell}}{H}}, \tag{13}$$

so that the lateral energy of a single cell reads

$$W_{\rm l} = \Gamma_{\rm l}\frac{1}{\sqrt{1+C_1C_2H^2/12}}\sqrt{\frac{V_{\rm cell}}{H}}\left[\sqrt{\nu}\sqrt{H^2 + \frac{1}{\nu}\frac{(C_2H/2)^2}{1+C_1C_2H^2/12}\frac{V_{\rm cell}}{H}} + \frac{1}{\sqrt{\nu}}\sqrt{H^2 + \nu\frac{(C_1H/2)^2}{1+C_1C_2H^2/12}\frac{V_{\rm cell}}{H}}\right]. \tag{14}$$

By comparing this expression with $({\rm d}W_{\rm l}/{\rm d}A_{\rm m})\,\Delta A_{\rm m}$ and expressing $\Delta A_{\rm m}$ of the cross-section of the single cell as

$$\Delta A_{\rm m} = \frac{1}{1+C_1C_2H^2/12}\frac{V_{\rm cell}}{H}, \tag{15}$$

we find that

$$\begin{aligned} \frac{{\rm d}W_{\rm l}}{{\rm d}A_{\rm m}} &= \Gamma_{\rm l}\sqrt{1+\frac{1}{12}C_1C_2H^2}\sqrt{\frac{H^3}{V_{\rm cell}}} \\ &\times\left[\sqrt{\nu}\sqrt{1+\frac{1}{\nu}\frac{1}{1+C_1C_2H^2/12}\frac{C_2^2V_{\rm cell}}{4H}} + \frac{1}{\sqrt{\nu}}\sqrt{1+\nu\frac{1}{1+C_1C_2H^2/12}\frac{C_1^2V_{\rm cell}}{4H}}\right]. \end{aligned} \tag{16}$$

Upon inserting this result along with Eq. (7) into Eq. (6), we obtain the final energy density of a tissue section of midplane area $\Delta A_{\rm m}$. To recast it in dimensionless form, we divide it by $\Gamma_{\rm l} V_{\rm cell}^{2/3}$ and introduce the dimensionless energy $w = W/(\Gamma_{\rm l} V_{\rm cell}^{2/3})$, dimensionless midplane area $A = A_{\rm m}/V_{\rm cell}^{2/3}$, dimensionless cell height $h = H/V_{\rm cell}^{1/3}$, dimensionless curvatures $c_1 = C_1 V_{\rm cell}^{1/3}$ and $c_2 = C_2 V_{\rm cell}^{1/3}$, dimensionless apical tension $\alpha = \Gamma_{\rm a}/\Gamma_{\rm l}$, and dimensionless basal tension $\beta = \Gamma_{\rm b}/\Gamma_{\rm l}$. Upon taking the limit $\Delta A_{\rm m} \to 0$, we find that the final expression for the dimensionless energy per unit dimensionless area is

$$\begin{aligned}\frac{{\rm d}w}{{\rm d}A} &= (\alpha+\beta)\left(1+\frac{c_1c_2h^2}{4}\right)+(\beta-\alpha)\frac{(c_1+c_2)h}{2} \\ &+\sqrt{1+\frac{1}{12}c_1c_2h^2}\sqrt{h^3}\left[\sqrt{\nu}\sqrt{1+\frac{1}{\nu}\frac{1}{1+c_1c_2h^2/12}\frac{c_2^2}{4h}}+\frac{1}{\sqrt{\nu}}\sqrt{1+\nu\frac{1}{1+c_1c_2h^2/12}\frac{c_1^2}{4h}}\right]. \qquad (17)\end{aligned}$$

This result is rather involved but it can be considerably simplified if $c_1^2/4h$, $c_2^2/4h$, and $c_1c_2h^2/12$ are all considerably smaller than unity. (For example, the mean values of $c^2/4h$ and $c^2h^2/12$ in the epithelial shells in Figs. 3a and b of the main text are 0.026 and 0.025, respectively.) In this limit we find that

$$\begin{aligned}\frac{{\rm d}w}{{\rm d}A} &= (\alpha+\beta)\left(1+\frac{c_1c_2h^2}{4}\right)+(\beta-\alpha)\frac{(c_1+c_2)h}{2} \\ &+\left(\sqrt{\nu}+\frac{1}{\sqrt{\nu}}\right)h^{3/2}+\frac{1}{24}\left(\sqrt{\nu}+\frac{1}{\sqrt{\nu}}\right)c_1c_2h^{7/2}+\frac{1}{8}h^{1/2}\left(\sqrt{\nu}c_1^2+\frac{1}{\sqrt{\nu}}c_2^2\right). \qquad (18)\end{aligned}$$

Note that the form of Eq. (18) is also obtained in the limit of small deformations where $c_1h \to 0$ and $c_2h \to 0$; this limit is however less relevant for our epithelial shells.

We now further assume that the tissue is fluidized so that $\nu = 1$, and we rearrange the above expression as

$$\begin{aligned}\frac{{\rm d}w}{{\rm d}A} &= \underbrace{(\alpha+\beta)+2\left[1-\frac{(\alpha-\beta)^2}{4}\right]\sqrt{h^3}}_{\text{surface tension}} \\ &\quad \underbrace{+\frac{\sqrt{h}}{8}\left[c_1+c_2-2\sqrt{h}(\alpha-\beta)\right]^2}_{\text{local bending energy}} \\ &\quad \underbrace{+\left(\frac{\alpha+\beta}{4}+\frac{\sqrt{h^3}}{12}-\frac{1}{4\sqrt{h^3}}\right)h^2c_1c_2}_{\text{Gaussian bending energy}}. \qquad (19)\end{aligned}$$

The first term, which is independent of curvature, represents the surface tension of the tissue, the second one is the local bending energy with a non-zero spontaneous curvature analogous to the bending energy of lipid membranes within the spontaneous-curvature model [1], whereas the third term is the Gaussian bending energy. The analogy with lipid membranes is not complete as all three terms also depend on the local tissue thickness h; in lipid membranes, thickness is assumed to be constant.

To estimate the local bending modulus $k_{\rm c}$, the Gaussian modulus $k_{\rm G}$, and the spontaneous curvature c_0, we replace the actual value of h (which is a variable rather than a constant) by the equilibrium cell height corresponding to a flat epithelium, which reads $h = (\alpha+\beta)^{2/3}$; here we assumed that the apical and the basal sides are squares. This gives

$$k_{\rm c} = \frac{1}{4}(\alpha+\beta)^{1/3}, \qquad (20)$$

$$k_{\rm G} = \left[\frac{1}{3}(\alpha+\beta)^2-\frac{1}{4}\right](\alpha+\beta)^{1/3}, \qquad (21)$$

and

$$c_0 = 2(\alpha+\beta)^{1/3}(\alpha-\beta) \qquad (22)$$

The curvature-thickness coupling term $(\beta-\alpha)(c_1+c_2)h/2$ in Eq. (18), which gives rise to the spontaneous curvature in Eq. (19), can also be interpreted in a different manner. We again consider a fluidized tissue with $\nu=1$ and we assume that the tissue forms a closed shell as in the main text. We then integrate the energy per unit area over the entire surface, which gives

$$w=\oint_A\left[(\alpha+\beta)\left(1+\frac{c_1c_2h^2}{4}\right)+2h^{3/2}+\frac{1}{12}c_1c_2h^{7/2}+\frac{1}{8}h^{1/2}\left(c_1^2+c_2^2\right)\right]\mathrm{d}A+\frac{\beta-\alpha}{2}\oint_A(c_1+c_2)h\,\mathrm{d}A. \tag{23}$$

If h does not vary across the shell and is small compared to the size of the shell, then the integral $\oint_A(c_1+c_2)h\,\mathrm{d}A$ is equal to the difference between the apical and the basal area of the shell ΔA, and we have

$$w\ \ =f(\alpha+\beta)+\tfrac{1}{2}(\beta-\alpha)\Delta A, \tag{24}$$

where f is a shape-dependent function of total tension $\alpha+\beta$ but not of differential tension $\beta-\alpha$. From the form of this expression we see that the differential tension $\beta-\alpha$ is coupled to ΔA and thus $(\beta-\alpha)/2$ effectively acts as an area-difference tension. In this respect, the continuum theory of our model tissues derived above departs from the area-difference-elasticity theory of lipid vesicles where the so-called non-local bending energy gives rise to a term $\propto(\Delta A-\Delta A_0)^2$ [3].

As an estimate of the error arising from rectangular rather than a hexagonal base, our continuum theory gives the height of a flat epithelium as $(\alpha+\beta)^{2/3}$, whereas the exact result for a flat epithelium of hexagonal cells is $\left(2^{1/3}/3^{1/6}\right)(\alpha+\beta)^{2/3}$, a difference of only about 5%; presumably, a similar relatively small scale of error can be assumed for c_1 and c_2, and all terms are higher than first order.

Supplementary References

[1] Seifert, U., Berndl, K. & Lipowsky, R. Shape transformation of vesicles: Phase diagram for spontaneous-curvature and bilayer-coupling models. *Phys. Rev. A* **44**, 1182–1202 (1991).

[2] Krajnc, M. & Ziherl, P. Theory of epithelial elasticity. *Phys. Rev. E* **92**, 052713 (2015).

[3] Svetina, S. & Žekš, B. Membrane bending energy and shape determination of phospholipid vesicles and red blood cells. *Eur. Biophys. J.* **17**, 101–111 (1989).
