# Supplementary material for: Collective cell mechanics of epithelial shells with organoid-like morphologies
Source: Nat Commun. 2020 Jul 30;11:3805. doi: 10.1038/s41467-020-17535-4 (PMC7393134; doi:10.1038/s41467-020-17535-4)
Supplement: Supplementary file 7 — Description of Additional Supplementary Files [file 41467_2020_17535_MOESM7_ESM.pdf]

**Description of Additional Supplementary Files**

File Name: Supplementary Movie 1
Description: Evolution of shapes of the three model epithelial shells in Fig. 1c of the main text without active T1 transitions ($k_{\mathrm{T1}}^{(0)}$ = 0), $N_c$ = 300, and $v_{\mathrm{lumen}}$ = 100. The simulations start from a spherical shape and result in a spherical (α = 1.2, β = 1.2), stomatocyte (α = 0.5, β = 1.1), and budded (α = 1.5, β = 0.3) morphology.

File Name: Supplementary Movie 2
Description: Evolution of shapes of the four active T1 model epithelial shells in Fig. 1d of the main text, with $k_{\mathrm{T1}}^{(0)}$ = 200, $N_c$ = 300, and $v_{\mathrm{lumen}}$ = 100. The simulations start from a spherical shape and result in a spherical (α = 1.2, β = 1.2), stomatocyte (α = 0.5, β = 1.1), budded (α = 1.1, β = 0.5), and branched (α = 0.7, β = 0.5) morphology.

File Name: Supplementary Movie 3
Description: Evolution of the branched model epithelial shell in Fig. 2d of the main text (α = 0.7, β = 0.5). Cells with five neighbors are colored yellow, cells with seven neighbors are colored brown, and all other cells are white.

File Name: Supplementary Movie 4
Description: Evolution of shapes of the four growing model epithelial shells with $\tau_{\mathrm{d}}$ = 2000 in Figs. 4b-e of the main text. Note that while in the spherical (α = 1.2, β = 1.2), stomatocyte (α = 0.5, β = 1.1), budded (α = 1.1, β = 0.5) epithelial shells the shapes resulting from growth and from junctional activity (Fig. 1d of the main text) belong to the same category, the (α = 0.7, β = 0.5) growth-induced shape differs from the branched morphology in Fig. 1d as branching requires the presence of active T1 transitions, which are absent in the growing-shell model. u

File Name: Supplementary Movie 5
Description: Evolution of shapes of the two growing model epithelial shells with $\tau_{\mathrm{d}}$ = 2000 in Figs. 4f-g of the main text with (α = 0.7, β = 0.5), without active T1 transitions ($k_{\mathrm{T1}}^{(0)}$ = 0; left), and with active T1 transitions ($k_{\mathrm{T1}}^{(0)}$= 200; right).
