# Supplementary material for: Collective cell mechanics of epithelial shells with organoid-like morphologies
Source: Nat Commun. 2020 Jul 30;11:3805. doi: 10.1038/s41467-020-17535-4 (PMC7393134; doi:10.1038/s41467-020-17535-4)
Supplement: Supplementary file 8 — Reporting Summary [file 41467_2020_17535_MOESM8_ESM.pdf]

nature research

Corresponding author(s): Jan Rozman

Last updated by author(s): Jun 13, 2020

Reporting Summary

Nature Research wishes to improve the reproducibility of the work that we publish. This form provides structure for consistency and transparency in reporting. For further information on Nature Research policies, see our Editorial Policies and the Editorial Policy Checklist.

Statistics

For all statistical analyses, confirm that the following items are present in the figure legend, table legend, main text, or Methods section.

n/a	Confirmed	
[x]	[ ]	The exact sample size (n) for each experimental group/condition, given as a discrete number and unit of measurement
[x]	[ ]	A statement on whether measurements were taken from distinct samples or whether the same sample was measured repeatedly
[x]	[ ]	The statistical test(s) used AND whether they are one- or two-sided<br>*Only common tests should be described solely by name; describe more complex techniques in the Methods section.*
[x]	[ ]	A description of all covariates tested
[x]	[ ]	A description of any assumptions or corrections, such as tests of normality and adjustment for multiple comparisons
[ ]	[x]	A full description of the statistical parameters including central tendency (e.g. means) or other basic estimates (e.g. regression coefficient) AND variation (e.g. standard deviation) or associated estimates of uncertainty (e.g. confidence intervals)
[x]	[ ]	For null hypothesis testing, the test statistic (e.g. F, t, r) with confidence intervals, effect sizes, degrees of freedom and P value noted<br>*Give P values as exact values whenever suitable.*
[x]	[ ]	For Bayesian analysis, information on the choice of priors and Markov chain Monte Carlo settings
[x]	[ ]	For hierarchical and complex designs, identification of the appropriate level for tests and full reporting of outcomes
[x]	[ ]	Estimates of effect sizes (e.g. Cohen's d, Pearson's r), indicating how they were calculated

Our web collection on statistics for biologists contains articles on many of the points above.

Software and code

Policy information about availability of computer code

Data collection	The simulated epithelial-shell shapes were computed using custom code written in C++ programing language (C++11).
Data analysis	Data analysis was conducted using custom code written in C++ programing language (C++11) and Wolfram Mathematica (version 10.3).

For manuscripts utilizing custom algorithms or software that are central to the research but not yet described in published literature, software must be made available to editors and reviewers. We strongly encourage code deposition in a community repository (e.g. GitHub). See the Nature Research guidelines for submitting code & software for further information.

Data

Policy information about availability of data

All manuscripts must include a data availability statement. This statement should provide the following information, where applicable:

- Accession codes, unique identifiers, or web links for publicly available datasets
- A list of figures that have associated raw data
- A description of any restrictions on data availability

The data containing the computed epithelial-shell shapes is available from the corresponding author on reasonable request.

Field-specific reporting

Life sciences study design

All studies must disclose on these points even when the disclosure is negative.

Sample size	Where statistical averaging was required, each simulation was run at multiple seeds for the random number generator, and the results were averaged over these runs; the number of different seeds is specified for each case in the manuscript.
Data exclusions	As described in the manuscript, the non-physical model epithelial-shell shapes at low tensions were excluded from the analysis. This applies to all the computed shapes that serve as the raw data in the manuscript, including both the relaxed shells (Fig. 1 through Fig. 3) as well as the growing shells (Fig. 4). When preparing the data for analysis, we automatically checked for self-overlap in each individual computed shell shape using pre-established algorithm implemented in Wolfram Mathematica (version 10.3).
Replication	To ensure reproducibility of results we described all details of the model and provided the values of all model parameters.
Randomization	This is a theoretical study so this does not apply.
Blinding	This is a theoretical study so this does not apply.

Reporting for specific materials, systems and methods

We require information from authors about some types of materials, experimental systems and methods used in many studies. Here, indicate whether each material, system or method listed is relevant to your study. If you are not sure if a list item applies to your research, read the appropriate section before selecting a response.

Materials & experimental systems

n/a	Involved in the study	
☒	☐	Antibodies
☒	☐	Eukaryotic cell lines
☒	☐	Palaeontology and archaeology
☒	☐	Animals and other organisms
☒	☐	Human research participants
☒	☐	Clinical data
☒	☐	Dual use research of concern

Methods

n/a	Involved in the study	
☒	☐	ChIP-seq
☒	☐	Flow cytometry
☒	☐	MRI-based neuroimaging
